# International Brazilian Journal of Urology Is the Official Information Journal of the American Confederation of Urology - CAU

**DOI:** 10.1590/S1677-5538.IBJU.2021.02.01

**Published:** 2021-02-03

**Affiliations:** 1 Universidade do Estado de Rio de Janeiro Unidade de Pesquisa Urogenital Rio de Janeiro RJ Brasil Unidade de Pesquisa Urogenital - Universidade do Estado de Rio de Janeiro - Uerj, Rio de Janeiro, RJ, Brasil.; 2 Hospital Federal da Lagoa Rio de Janeiro RJ Brasil Serviço de Urologia, Hospital Federal da Lagoa, Rio de Janeiro, RJ, Brasil.

We are pleased to announce that International Brazilian Journal of Urology became the official communication organ of the American Confederation of Urology. The editorial board and the Brazilian Urology Society are very happy because the beginning of this partnership what will contribute to reinforce the impact of the International Brazilian Journal of Urology on the American continent.

We are experiencing very difficult times because of the Covid-19 pandemic and at the time of writing this editorial a second wave of the disease is starting in Europe. However, research cannot stop. The March-April 2021 number of Int Braz J Urol, the ninth under my supervision, presents original contributions with a lot of interesting papers in different fields: Prostate Cancer, Urethral Stricture, Male Incontinence, Vesicoureteral Reflux, Renal Cell Carcinoma, Bladder Cancer, BPH, SARS-CoV-2 and Urology, Radiation Induced Cystitis, Vaginoplasty, Varicocele, Basic Research applied to Female Urinary Incontinence, Flexible Ureteroscopy, Penile Fracture and Straghorn Renal Calculi. The papers came from many different countries such as Brazil, USA, Turkey, China, Iran, Portugal, Serbia, Montenegro and Spain, and as usual the editor's comment highlights some of them.

In the present issue we present four important papers in reconstructive urology and 2 very important papers about SARS-CoV-2 pandemic in Urology. Dr. Benson and colleagues from USA performed in page 237 (1) a nice sistematic review about the long term outcomes of one-stage augmentation anterior urethroplasty and concluded that the long-term success rates of augmentation urethroplasty are appear to be worse than previously appreciated and patients should be counseled accordingly. Dr. Barros and collegues (2) from Rio de Janeiro - Brazil performed in page 389 a important paper about a rare condition: The penile fracture. In this paper the authors described penile fracture (PF) findings with non-sexual etiology in a referral emergency hospital, with emphasis on demographic data, clinical and intraoperative findings and long-term outcomes and concluded that PF is a rare urologic emergency, especially with the non-sexual etiology. However, PF should always be considered when the clinical presentation is suggestive, regardless of the etiology. Penile manipulation and roll over in bed were the most common non-sexual causes. These cases are related to low-energy traumas, usually leading to unilateral rupture of corpus cavernosum. Urethral involvement is uncommon but may be present. Early treatment has good long-term clinical outcome, especially when performed in specialized centers with extensive experience in FP.

Dr. Angulo and colleagues from Spain and Portugal (3) present in page 399 an important study about urorectal fistula approaches and concluded that the surgical approach elected to correct URF is not determinant of success nor of complications. Fistula size appears as independent determinant for failure. Trans-sphincteric approach could be advantageous over other procedures regarding HRQoL issues and the last paper about reconstructive urology is performed by the group of Dr. Allen Moorey (4) in page 415. The authors studied the standing cough test stratification of moderate male stress urinary incontinence and concluded that many men with self-reported history of moderate SUI actually present severe SUI observed on SCT. The SCT is a useful tool to stratify moderate SUI patients to more accurately predict sling success.

The 2 papers about COVID-19 in urology came from Brazil. Dr. Mazzuchi and collegues from Brazil presented in page 251 (5) a nice review about the impact of COVID-19 in medical practice focused on Urology and concluded that despite not been a urological disease, the urologist needs to be updated on how to deal with these patients and how to take care of himself and of the medical team he works with the disease. Dr. Silva and collegues from Brazil presented in page 378 (6) an important study about the evaluation of uro-oncological surgical treatment during the Sars-CoV-2 pandemic in a Brazil and concluded that elective uro-oncological procedures at the COVID-19 epidemic period in a COVID-19-free Institute are safe, and patients who need urgent procedures, with a long period of hospitalization, need special care to avoid COVID-19 infection and its outcomes. The editor in chief would like to highlight the following works too:

Dr. Jeremias and collegues from Brazil (7) on page 275 evaluate the oxidative origin of sperm DNA fragmentation in the adult varicocele and concluded that patients with varicocele have an increase in sperm DNA fragmentation levels, particularly in oxidative stress-induced sperm DNA damage.

Dr. Castellanti de Mattos and collegues from Brazil (8) performed on page 295 a very interesting study about the evaluation of HIF-1a and VEGF-A expression in radiation- induced cystitis. This study is on the cover in this number. The authors concluded that despite the lack of statistical significance precludes a definitive conclusion, the data presented herein suggests that further studies investigating the role of HIF-1α in bladder neovascularization in radiation-induced cystitis are highly recommended.

Dr Menezes and collegues (9) from Brazil perfomed on page 308 a interesting anatomic study about the veromontonum and they described a new and interesting anatomic classification where they found five different verumontanum types: “volcano” (51.61%), “lighthouse” (24.73%), “whale tail” (12.90%), “hood” (5.38%) and “castle door” (5.38%) and concluded that Veromontanum has smaller measurements in patients with BPH regardless of treatment. In the control group, there was an increase in verumontanum diameters with an increase in BMI. The volcano type of verumontanum was the most frequent regardless of groups and BMI.

Dr. Wang and collegues (10) from China proposed on page 333 a study to establish and evaluate a nomogram for predicting the overall survival (OS) and cancer-specific survival (CSS) of RCC patients with bone metastasis and concluded that the nomograms for predicting prognosis provided an accurate prediction of OS and CSS in RCC patients with bone metastasis, and contributed clinicians to optimize individualized treatment plans.

Dr. Loftus and collegues (11) from USA investigated on page 350 the effect of various intra-renal pressures on histologic changes and fluid extravasation during simulated ureteroscopy and concluded that pressurized endoscopic irrigation leads to significant extravasation of fluid into the renal parenchyma. Higher intra-renal pressures were associated with increased penetration of irrigant during ureteroscopy in an ex-vivo porcine model.

Dr. Maluf and collegues (12) from Brazil shows on page 359 and interesting consensus about the non-metastatic castration resistant prostate cancer (M0 CRPC) in Brazil and concluded that the use of apalutamide and enzalutamide in patients with M0 CRPC who were not eligible for PROSPER or SPARTAN due to clinical and laboratory factors, such as shorter life expectancy and longer PSAtd, with the goal to facilitate clinical decision making

Drs. Djordjevic and Vokovic (13) from Serbia and Montenegro shows on page 426 a interesting surgical technique about the modified Hautmann neobladder with Wallace ureteroileal anastomosis on a 6-8 cm long isoperistaltic chimney, following radical cystectomy and concluded that the functional results with modified Hautmann neobladder, incorporating short afferent limb in Wallace I uretero-enteric anastomosis, were efficient. This technique is an effective way to minimize potential uretero-enteric stricture, anastomotic leakage and incidence of vesicoureteral reflux.

We hope that readers will enjoy the present number of the International Brazilian Journal of Urology in this very difficult times of COVID-19.
